# When Sepsis Is Not Sepsis: A Case Report of Autoimmune Myopathy Masquerading as Postoperative Infection

**DOI:** 10.7759/cureus.91807

**Published:** 2025-09-07

**Authors:** Sharlet Shabu Pappachan, Rizana Riyaz, Jane Snook, Anush Nagotu, Mohammed Nazim Kunduvalappil Thachankandy

**Affiliations:** 1 Geriatrics, Princess Alexandra Hospital NHS Trust, Harlow, GBR; 2 Molecular Medicine, University of Sheffield, Sheffield, GBR; 3 General Surgery, Lister Hospital, Stevenage, GBR; 4 Geriatrics, Royal Berkshire Hospital, Reading, GBR

**Keywords:** corticosteroid therapy, hip hemiarthroplasty, postoperative complications, postoperative pyrexia, statin-induced necrotising autoimmune myopathy

## Abstract

A 79-year-old male with multiple comorbidities underwent left hemiarthroplasty following a neck of femur fracture. Necrotizing autoimmune myopathy (NAM), a rare idiopathic inflammatory myopathy that can mimic postoperative sepsis and lead to diagnostic delays, complicated his postoperative course. From the first day after surgery, he experienced persistent pyrexia with markedly elevated inflammatory markers. Despite extensive septic screening and broad-spectrum antimicrobials, all microbiological investigations remained negative, and the patient continued to deteriorate. Nine days postoperatively, he developed severe pain and progressive symmetrical weakness, rapidly evolving into quadriparesis with significantly reduced muscle power in all limbs and elevated creatine kinase levels. A comprehensive myositis-specific antibody panel was entirely negative, despite the strong clinical suspicion for idiopathic inflammatory myopathy. Given the clinical deterioration and negative infectious workup, empirical low-dose corticosteroid therapy was initiated. The patient demonstrated dramatic clinical improvement within days of steroid initiation. Temperature spikes ceased, muscle power rapidly improved in all limbs within 10 days, and inflammatory markers significantly decreased. The patient progressed from bedridden to mobilising with a mobility aid within two weeks. This case highlights the diagnostic challenge of distinguishing NAM from postoperative sepsis. The seronegative presentation emphasises the importance of clinical acumen when conventional serological markers are absent. Early recognition and corticosteroid therapy are crucial for optimal outcomes in statin-associated NAM. Clinicians should maintain high suspicion for autoimmune myopathy in patients with unexplained postoperative weakness and negative infectious investigations.

## Introduction

Necrotizing autoimmune myopathy (NAM) is said to affect 1.85 per 100,000 individuals over age 50; its ability to masquerade as postoperative sepsis transforms this rare entity into a diagnostic nightmare [1]. This reality was starkly demonstrated when a 79-year-old man, following routine hip surgery, had a refractory fever and progressive quadriparesis, which led to a critical diagnostic delay that could have proven devastating in the face of rapidly progressive muscle destruction.

NAM is a subgroup of idiopathic inflammatory myopathies, characterized by myocyte necrosis without significant inflammation, likely immune-mediated and responding to immunotherapy [2]. The European Neuromuscular Centre International diagnostic criteria for acute necrotising inflammatory myopathy include subacute muscle weakness, elevated creatine kinase (CK) levels, abnormal electromyography (EMG), and necrotic muscle fibres with minimal or no inflammatory infiltrates [3].

NAM is associated with statin administration and is most often related to cancer or connective tissue disorders [4]. With a median age of onset at 64 years, clinically, it presents with lower limb weakness, distal limb weakness, dysphagia, and/or dyspnoea [1,4]. They can be diagnosed with serum anti-signal recognition particle (SRP) and anti-hydroxy-3-methylglutaryl-CoA reductase (HMGCR) autoantibodies [5]. NAM/immune-mediated necrotising myopathy (IMNM) has been subclassified to include anti-SRP-positive, anti-HMGCR-positive, and seronegative IMNM [6].

IMNMs are severe and progressive if left untreated. With treatment, outcomes are usually positive; adverse outcomes or fatalities can occur, either due to delayed intervention, inadequate therapy, cancer-related causes, or cardiorespiratory complications associated with IMNM [7]. We report a diagnostically challenging case of acute inflammatory myopathy in a 79-year-old male, which mimicked postoperative sepsis.

## Case presentation

Written informed consent for publication of this case report was obtained from the patient. This case report was structured according to the CARE (CAse REport) guidelines to ensure comprehensive and transparent reporting.

Patient information and initial management

A 79-year-old male with a significant past medical history presented to the emergency department on 08 May 2025 after a non-syncopal fall that resulted in a left neck of femur fracture. His medical history was notable for ischaemic heart disease with two coronary stents placed in 2013, heart failure with a reduced ejection fraction of 25%, and a permanent pacemaker implanted 9.5 years prior. He also had chronic obstructive pulmonary disease (COPD), a history of left partial pneumonectomy in 2018, and gout. On 10 May 2025, he underwent a left hemiarthroplasty. Following regular preoperative protocol, some of his medications, including statins, were suspended.

Clinical findings and postoperative course

The patient’s postoperative course was immediately complicated by pyrexia (moderate grade - intermittent), which began on postoperative day one (11 May 2025). An extensive septic screen was initiated. Initial laboratory findings revealed a C-reactive protein (CRP) of 411 mg/L and a procalcitonin of 0.6 ng/mL. However, two blood cultures, urine microscopy culture and sensitivity (MCS), and a viral PCR screen were all negative.

**Figure 1:**
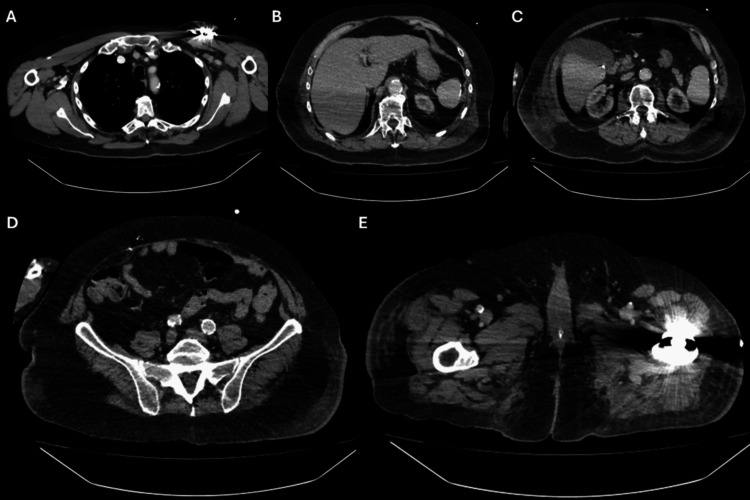
Axial contrast-enhanced computed tomography (CT) images. (A) Chest at the level of the thoracic inlet, with artefact from a right-sided pacemaker overlying the chest wall. (B) Upper abdomen. (C) Abdomen at the level of the renal hila. (D) Lower abdomen and pelvis. (E) Pelvis demonstrating bilateral hip prostheses with associated streak artefacts.

**Figure 2:**
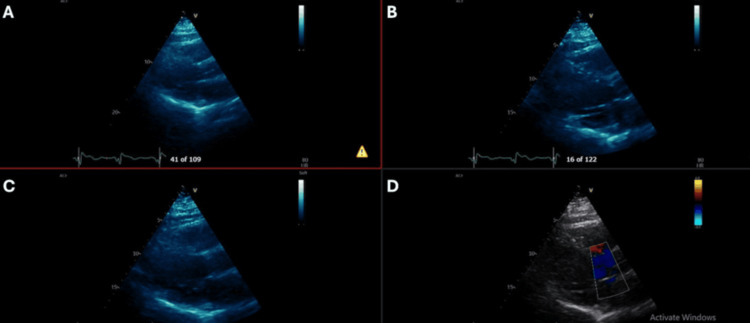
Transthoracic echocardiographic images. (A) Parasternal long-axis view. (B) Parasternal short-axis view. (C) Apical four-chamber view. (D) Color Doppler echocardiographic image demonstrating blood flow across the cardiac chambers. The transthoracic echocardiogram demonstrates a mildly dilated left ventricle (LV) with severely impaired systolic function (estimated ejection fraction 25-30%). Right ventricular (RV) size is normal, though RV function is reduced. Atrial sizes are within normal limits. Mild tricuspid regurgitation (TR) is present with intermediate probability of pulmonary hypertension. The aortic, tricuspid, and pulmonary valves appear structurally normal, with no evidence of stenosis. The aortic root and main pulmonary artery are of normal dimension. The pericardium is intact with no pericardial effusion. The interatrial and interventricular septa appear intact, with no abnormal color Doppler flow. The inferior vena cava (IVC) shows normal inspiratory variability (>50%).

The fever persisted despite empirical escalation of antibiotics to teicoplanin and meropenem, which were chosen due to the patient's penicillin allergy. A comprehensive search for an infectious source was unrevealing; a CT of the chest, abdomen, and pelvis (Figure 1) showed no evidence of active infection or malignancy, and an echocardiogram (Figure 2) ruled out infective endocarditis. Repeat blood cultures remained negative. The patient failed to respond to broad-spectrum antimicrobials, arguing against an infectious aetiology.

The extended workup was performed to exclude infectious and other autoimmune causes of the patient's fever and systemic inflammation, as in Table 1. All results supported a non-infectious, non-vasculitic aetiology, contributing to the diagnostic shift toward inflammatory myopathy.

**Table 1:** Extended infectious disease and immunological investigations. Ag = Antigen; CMV = Cytomegalovirus; EBV = Epstein-Barr Virus; PCR = Polymerase Chain Reaction; abs = Antibodies; ANCA = Anti-neutrophil Cytoplasmic Antibodies; Anti CCP = Anti-Cyclic Citrullinated Peptide Antibodies; cr = Creatinine

Test Name	Result	Normal Range	Interpretation
Legionella urine Ag	Negative	Negative	Normal - excludes *Legionella pneumonia*
CMV IgG	Positive	Negative	Past CMV infection (not active)
CMV IgM	Negative	Negative	Normal - excludes acute CMV infection
EBV DNA PCR	Negative	Negative	Normal - excludes active EBV infection
Heterophile abs	Negative	Negative	Normal - excludes infectious mononucleosis
ANCA	Negative	Negative	Normal - excludes ANCA-associated vasculitis
Anti CCP	0.6 U/mL	<3.0 U/mL	Normal - excludes rheumatoid arthritis
Urine protein creatinine ratio	72 mg/mmol cr	<30 mg/mmol cr	Mildly elevated - likely secondary to systemic inflammation

Beginning on 16 May 2025, approximately eight days postsurgery, the patient's clinical picture changed dramatically. He developed severe pain and progressive weakness, initially presenting as symmetrical proximal weakness, first noted in the left upper limb, which rapidly evolved into a quadriparesis affecting all four limbs by 20 May 2025. This weakness was accompanied by marked muscle tenderness, severe myalgia, and allodynia. Physical examination revealed muscle power 1/5 in all four limbs, with proximal muscles more severely affected than distal. There were no cutaneous manifestations or signs of joint inflammation.

Diagnostic assessment

The initial diagnostic focus was on postoperative sepsis, given the patient's fever, high inflammatory markers, and surgical context. However, this diagnosis became untenable due to the persistently negative microbiological and imaging investigations and a decreasing procalcitonin level (from 0.6 to 0.3 ng/mL), which argued against a bacterial cause.

The emergence of progressive, symmetrical, proximal-dominant weakness, severe myalgia, and marked muscle tenderness prompted a shift in the diagnostic approach. Key laboratory findings supporting a myopathic process included a fluctuating but significantly elevated CK, which was 1,081 U/L initially postop, which came down to 300 U/L and increased to 997 U/L on 16 May 2025. In addition, inflammatory markers remained extremely high, with the CRP rising to 493 mg/L and the erythrocyte sedimentation rate (ESR) reaching 129 mm/hour, as shown in Table 2.

**Table 2:** Serial CRP, CK, and ESR measurements during the course of hospitalization. This table displays the trends in the patient's creatine kinase (CK), C-reactive protein (CRP), and erythrocyte sedimentation rate (ESR) from 8 May 2025 to 23 May 2025.

Dates	8 May 2025	12 May 2025	13 May 2025	15 May 2025	16 May 2025	18 May 2025	19 May 2025	20 May 2025	21 May 2025	23 May 2025	References
CK	1081	610	356	997	993	-	947	416	122	46	40-320 IU/L
CRP	43	411	388	410	460	493	461	447	304	130	0-5 mg/L
ESR	-	-	-	-	-	129	-	-	97	118	0-30 mm/hr

The symptoms and signs, including acute quadriparesis, severe myalgia, elevated CK and inflammatory markers, and a negative infectious workup, led to a working diagnosis of an idiopathic inflammatory myopathy (IIM). Consequently, a comprehensive myositis-specific antibody panel and an antinuclear antibody (ANA) test were sent for analysis. Additional immunological investigations were performed to exclude other autoimmune conditions, as shown in Table 3.

**Table 3:** Immunological investigation results. IgG = Immunoglobulin G; ACL Abs = Anti-cardiolipin Antibodies; GPL = IgG Phospholipid Units; MPL = IgM Phospholipid Units; Glom b Membrane Ab = Anti-glomerular Basement Membrane Antibodies

Test Name	Result	Normal Range	Interpretation
IgG1	5.19 g/L	3.20-10.20 g/L	Normal
IgG2	2.15 g/L	1.20-6.60 g/L	Normal
IgG3	0.24 g/L	0.20-1.90 g/L	Normal
IgG4	0.36 g/L	0.00-1.30 g/L	Normal
ACL Abs (IgG)	5.5 GPL U/mL	0.00-12.00 GPL U/mL	Normal
ACL Abs (IgM)	3.10 MPL U/mL	0.00-9.40 MPL U/mL	Normal
Glom b membrane Ab	<1.5 U/mL	<7.00 U/mL	Normal

Therapeutic intervention

In the face of ongoing clinical deterioration and lack of a definitive non-infectious diagnosis, a therapeutic trial of steroids was initiated based on the strong clinical suspicion of an inflammatory myopathy. The patient was started on a modest dose of 20 mg oral prednisolone daily. This was administered concurrently with the ongoing broad-spectrum antibiotic coverage to ensure a potential occult infection was not missed while treating the suspected autoimmune process. As a precaution, medications with known myotoxic potential, including quinine sulphate, allopurinol, and fluconazole, were suspended when his symptoms of weakness began in his left upper limb. Statin therapy was discontinued prior to surgery and not resumed postoperatively. Figure 1 illustrates the timeline of clinical presentation and management of postoperative necrotising autoimmune myopathy.

Follow-up and outcomes

The patient demonstrated a dramatic clinical response to corticosteroid therapy. Within days of steroid initiation, his temperature spikes ceased, limb power began to improve rapidly, and severe pain subsided. Subsequently, there was a significant reduction in CRP and ESR levels, showing a dramatic downtrend, as shown in Table 4. Within two weeks, the patient, who had been bedridden and progressively deteriorating, could mobilize with a mobility aid (rollator frame).

**Table 4:** Clinical and laboratory response to corticosteroid therapy. CRP = C-Reactive Protein; ESR = Erythrocyte Sedimentation Rate; CK = Creatine Kinase This table demonstrates the dramatic clinical and biochemical response to corticosteroid therapy (prednisolone 20 mg daily) initiated based on suspected inflammatory myopathy. Power assessment used the Medical Research Council (MRC) scale, where 1/5 represents trace muscle contraction and 5/5 represents normal power against full resistance.

Parameter	Pre-steroids	Post-steroids	References
Pain	Severe, widespread	Improved by day 1	-
Temperature	Spiking fevers	Afebrile from day 2	-
Upper limb power	1/5	3/5 (day 3), 4/5 (day 7), 5/5 (day 10)	5/5
Lower limb power	1/5	5/5 (day 10)	5/5
CRP (mg/L)	493 (peak)	40 (day 3 post-steroids)	0-5 mg/L
ESR (mm/hr)	129	97 post-treatment	0-30 mm/hr
CK (U/L)	997	46 (day 3 post-steroids)	40-320 IU/L

The patient’s rapid response to corticosteroids, marked by improved muscle strength, resolution of systemic symptoms, and normalisation of inflammatory markers, allowed us to narrow the diagnosis from IIM to necrotising autoimmune myopathy. The myositis-specific antibody panel results helped us rule out the other types of IIM, further supporting this diagnosis, as shown in Table 5.

**Table 5:** Myositis-specific antibody panel results. SRP = Signal Recognition Particle; PM-Sc = Polymyositis-Scleroderma; SAE1 = Small Ubiquitin-Like Modifier Activating Enzyme; NXP2 = Nuclear Matrix Protein 2; MDA5 = Melanoma Differentiation-Associated Protein 5; TIF1γ = Transcriptional Intermediary Factor 1 Gamma; Mi-2 = Nucleosome Remodelling Deacetylase

Test Name	Result	Interpretation
Ro - 52 antibodies	Negative	Normal - excludes anti-Ro52-associated myositis
OJ antibodies	Negative	Normal - excludes antisynthetase syndrome
EJ antibodies	Negative	Normal - excludes antisynthetase syndrome
PL-12 antibodies	Negative	Normal - excludes antisynthetase syndrome
PL-7 antibodies	Negative	Normal - excludes antisynthetase syndrome
SRP antibodies	Negative	Normal - excludes anti-SRP myopathy
Jo-1 antibodies	Negative	Normal - excludes antisynthetase syndrome
PM-Sc175 antibodies	Negative	Normal - excludes PM/Scl overlap syndrome
PM-Sc1100 antibodies	Negative	Normal - excludes PM/Scl overlap syndrome
Ku antibodies	Negative	Normal - excludes anti-Ku myositis
SAE1 antibodies	Negative	Normal - excludes inclusion body myositis
NXP2 antibodies	Negative	Normal - excludes juvenile dermatomyositis
MDAS antibodies	Negative	Normal - excludes anti-MDA5 dermatomyositis
TIF1g antibodies	Negative	Normal - excludes cancer-associated dermatomyositis
Mi-2b antibodies	Negative	Normal - excludes classic dermatomyositis
Mi-2a antibodies	Negative	Normal - excludes classic dermatomyositis

**Figure 3:**
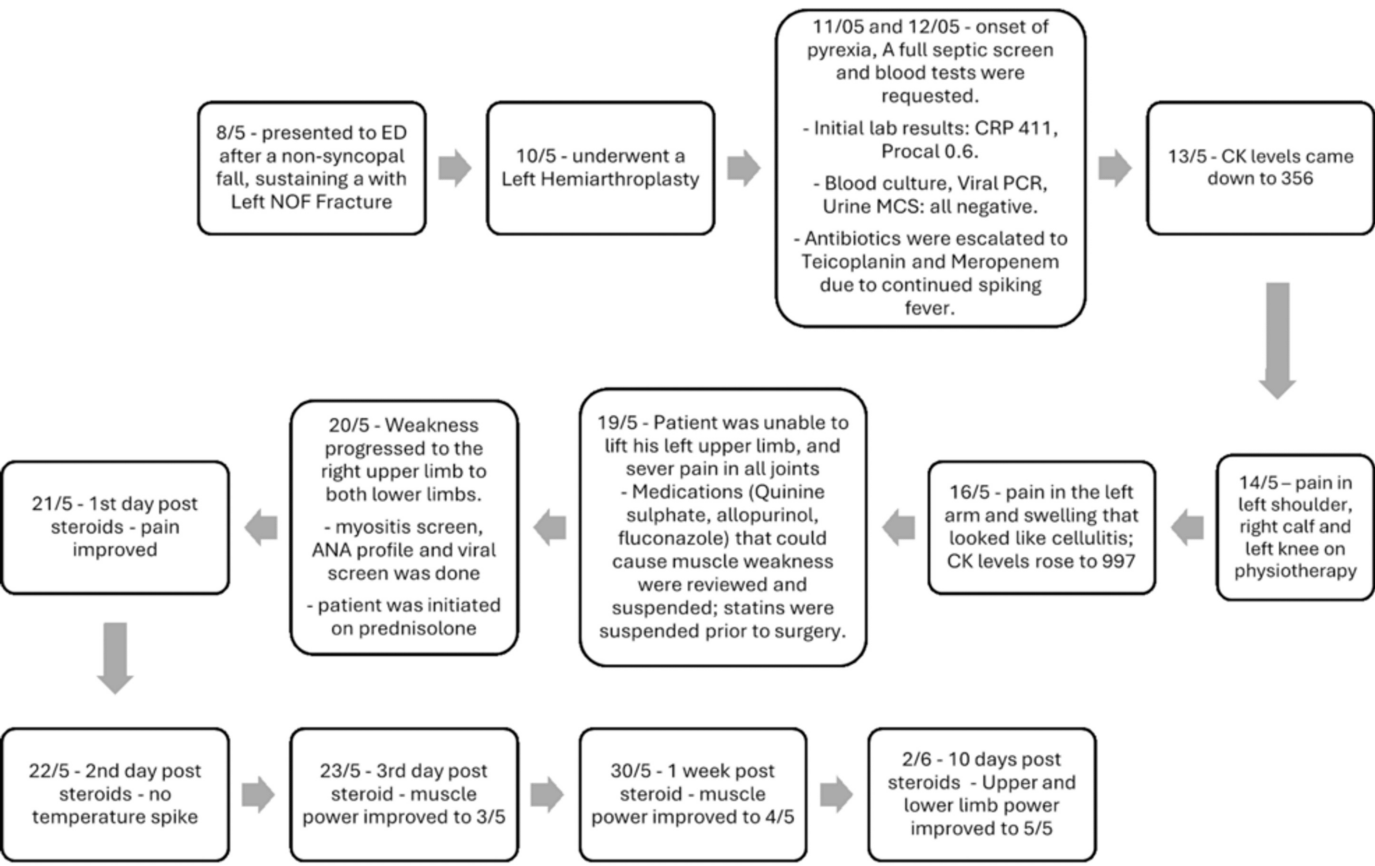
Timeline of clinical presentation and management of postoperative necrotizing autoimmune myopathy. Timeline depicting the clinical course of a 79-year-old male who developed necrotizing autoimmune myopathy following left hip hemiarthroplasty. Key events include initial surgery (day 0), onset of postoperative pyrexia (day 1), development of severe myalgia and weakness (days 8-9), progression to quadriparesis (day 12), initiation of prednisolone 20 mg daily (day 13), and subsequent clinical improvement with resolution of fever, recovery of muscle power, and normalization of inflammatory markers. CRP = C-Reactive Protein; CK = Creatine Kinase; ESR = Erythrocyte Sedimentation Rate

This comprehensive myositis-specific antibody panel was performed to identify specific autoimmune myopathy subtypes and guide targeted therapy. The entirely negative panel confirms a seronegative inflammatory myopathy. Notably, the absence of anti-SRP antibodies is commonly associated with statin use NAM, supporting the diagnosis of statin-induced NAM. Although the myositis screening, including SRP antibodies, was negative, there was a strong clinical suspicion of statin-induced NAM (SINAM) (Figure 3), given the patient’s history of high-dose atorvastatin (80 mg) use since 2013.

Following successful treatment, the patient is on a weaning corticosteroid regimen and is currently on 2 mg prednisolone daily and has achieved full recovery of his baseline mobility and functional capacity. Long-term monitoring for potential relapse will be essential, given that 55% of NAM patients experience disease relapse. Hence, further management by the rheumatologist will be required to monitor for potential relapse and guide long-term treatment.

## Discussion

This case demonstrates how a 79-year-old man with postoperative fever and profound weakness was initially managed as intractable sepsis but ultimately diagnosed clinically as SINAM. The diagnostic journey highlights the complexity of distinguishing between infectious and autoimmune aetiologies in postsurgical patients with systemic inflammation.

Postoperative sepsis was the initial working diagnosis due to the presence of fever (moderate grade, intermittent) and markedly elevated inflammatory markers, such as CRP > 400 mg/L and a functional decline following a hip surgery: A triad suggesting surgical site or systemic infection [8]. However, this diagnosis was systematically ruled out through: (1) repeatedly negative blood cultures (two sets), (2) negative viral PCR panels (as shown in Table 1), (3) negative urine cultures, (4) absence of vegetations on echocardiography, and (5) failure to respond to broad-spectrum antimicrobials. Subsequently, the patient developed progressive proximal limb weakness, followed by distal limb weakness, accompanied by severe myalgia, and was non-responsive to antimicrobial therapy. This demanded a paradigm shift in diagnostic approach. This prompted our differential diagnosis towards an immune aetiology, prompting investigations, as shown in Table 3.

Our focus was directed towards a group of autoimmune diseases known as the IIMs, as they are characterized by inflammation of the muscle (myositis) and other organ systems, resulting in widespread organ dysfunction, elevated morbidity, and early mortality. Accordingly, a comprehensive myositis antibody panel, encompassing a range of specific and associated autoantibodies, was performed; however, all results were negative, as shown in Table 5.

In contrast, biochemical markers told a different story: CK, a key myositis marker with established diagnostic and monitoring utility, was significantly elevated, as shown in Table 2, while ESR was markedly raised, indicating systemic inflammation. Coupled with the patient's clinical presentation of weakness and pain, these findings created a paradox. Despite negative serological markers, there was strong clinical and biochemical evidence supporting autoimmune myopathy, thus presenting a significant diagnostic conundrum.

As a result, in the face of ongoing deterioration and the lack of a definitive non-infectious diagnosis, a therapeutic trial of corticosteroids was initiated, commencing with a low dose of 20 mg oral prednisolone once daily, to which he responded well. The initial management of IIM typically involves corticosteroids, which are considered first-line agents due to their rapid onset and broad efficacy. The standard care often involves a starting dose of 1 mg/kg/day of prednisone or its equivalent, with high-dose pulsed methylprednisolone reserved for patients with severe or rapidly progressive disease involving dysphagia or respiratory compromise [7]. However, in this patient's case, the diagnostic uncertainty posed by potential concurrent sepsis necessitated a more cautious approach, as high-dose immunosuppression would have been detrimental had an infection been present. Consequently, a significantly lower initial dose of 20 mg oral prednisolone was administered, to which he responded well. The robust positive response to this low dosage is particularly noteworthy. It suggests that, in certain clinical scenarios, a lower-dose steroid regimen may be sufficient to control the autoimmune process, presenting a potential avenue for future clinical trials to minimize the well-known adverse effects associated with high-dose corticosteroid therapy.

While the clinical response was encouraging, establishing a definitive diagnosis remained crucial for long-term management. Conditions such as dermatomyositis, antisynthetase syndrome (ASS), toxic myopathies (TM), critical illness myopathy (CIM), and muscular dystrophy (MD) can mimic NAM. The following differential diagnoses were considered and systematically eliminated. Polymyositis is a rare IIM characterised by subacute-chronic limb weakness and increased creatine kinase values up to 50-fold in active disease. It was considered given the symmetrical proximal weakness and systemic inflammation [9]. However, it was deemed less likely because the CK level was not markedly raised, as is classic for the condition. Furthermore, the acute postoperative onset was atypical for its usual subacute progression. Diagnosing polymyositis relies on muscle biopsy, serum autoantibodies, EMG, and imaging techniques, with muscle histopathology being the key [10].

Secondly, dermatomyositis was considered due to its systemic nature affecting muscles and skin, with potential manifestations, including arthralgias, arthritis, oesophageal disease, and cardiopulmonary dysfunction. However, this diagnosis was excluded due to the absence of characteristic cutaneous findings, such as heliotrope rash or Gottron's papules. Finally, inclusion body myositis (IBM) was deemed unlikely given the acute presentation. IBM typically presents with a chronic, insidious course progressing over the years, causing significant disability from falls and grip weakness. It also commonly presents with weakness affecting the quadriceps, finger flexors or pharyngeal muscles, and ankylotic contractures impacting daily function. The dramatic response to corticosteroids argued strongly against IBM, a condition known for being steroid-resistant and unresponsive to immunotherapy [11].

Despite the negative myositis autoantibody screen, the compelling clinical picture and rapid response to low-dose corticosteroids were consistent with IIM. Due to the patient's prior statin exposure, we were able to arrive at the final diagnosis of SINAM. Although statins were discontinued preoperatively as per standard surgical protocols, this did not exclude the diagnosis of SINAM. SINAM has a well-documented association with statin therapy, with symptom onset potentially delayed up to 10 years after initiation or occurring months following discontinuation [12]. Given this wide spectrum of presentations, maintaining a high index of suspicion is crucial in patients with any history of statin use. While an association with malignancy exists, it is important to note that the overall cancer incidence in IMNM patients does not exceed that of the general population, and our CT imaging ruled out occult malignancies [1].

NAM represents a distinct autoimmune pathophysiology characterized by muscle necrosis, sparse inflammatory infiltrates, and complement deposition on muscle fibre membranes. Unlike typical statin-induced myalgia, NAM persists despite drug discontinuation and requires immunosuppressive therapy rather than simple cessation. The negative myositis autoantibody screen observed in this patient, as evidenced by Table 5, represents a recognized clinical phenomenon, as several patients with inflammatory myopathies have been reported to lack conventional myositis-specific antibodies [5,6]. Anti-HMGCR antibody testing was not performed, as it is not part of the routine myositis panel, and there was an urgent need to initiate corticosteroid therapy rather than delay treatment for a confirmatory diagnosis. In the context of an older, vulnerable patient, timely intervention was prioritised given the risk of rapid clinical deterioration. Furthermore, post-treatment testing may have yielded falsely low titres, reducing diagnostic accuracy, highlighting the importance of clinical correlation in geriatric care [13]. Furthermore, the identification of anti-HMG-CoA reductase antibodies carries significant prognostic implications, as patients who test positive should be carefully screened for malignancy due to an increased cancer risk observed in this population after age 50 and within three years of diagnosis [14]. However, as demonstrated by Kassardjian et al., who found the absence of SRP-IgG and/or HMGCR-IgG in 76% of patients, clinical judgment remains paramount in seronegative cases [4]. With respect to the available serological data, as shown in Table 4, anti-SRP antibodies, while typically present in NAM, are characteristically absent in statin-induced cases, as statins appear to have no association with the development of signal recognition particle (SRP)-IMNM. Therefore, this patient's negative anti-SRP result was consistent with statin-induced NAM, though the relationship between serum CK levels and anti-SRP autoantibodies in SRP-IMNM remains poorly understood [15].

Nevertheless, despite this serological gap, the compelling clinical picture, including prolonged statin exposure, characteristic symptoms, and excellent steroid response, supported the diagnosis. Considering the patient’s advanced age, prolonged hospital stay of over two weeks, and clear clinical response to corticosteroids, proceeding with a muscle biopsy was deemed inappropriate. In orthogeriatric care, a holistic approach is essential, balancing diagnostic yield against the risks of invasive procedures, including hospital-acquired infections and delayed recovery. In this context, clinical judgment took precedence to avoid additional burden on an already vulnerable patient.

Most affected patients require multiple immunotherapeutic agents, and Kassardjian et al. found that 55% of the patients who were treated relapsed, highlighting the importance of long-term monitoring in our patients [4]. Despite the clear diagnostic criteria for NAM, a significant clinical challenge is the median diagnostic delay of three months within a range of less than a month to 13 years [1].

This case illustrates how the significant physiological stress of major surgery may have triggered a pre-existing, subclinical autoimmune state. Although IMNM is typically linked to triggers such as viral infections and cancer, and a direct association with surgery has not been previously established, this case strongly suggests surgery can act as a precipitant, especially in patients with prior statin exposure [4,15,16]. This highlights several critical learning points for rheumatologists and general physicians. The initial presentation with prolonged fever and marked inflammatory markers postoperatively appropriately directed the diagnostic process towards infection. However, the consistently negative microbiological cultures, coupled with emerging signs of myopathy, including weakness, pain, and rising CK levels, necessitated a crucial paradigm shift in clinical thinking. This case also demonstrates that severe systemic inflammation, often clinically indistinguishable from sepsis on initial presentation, can represent the sole manifestation of an evolving autoimmune process.

The seronegative myositis screen presented a diagnostic challenge, underscoring the importance of clinical acumen and therapeutic response when serological markers are absent. This can be further complicated by the fact that patients with myositis-spectrum disorders, particularly cancer-associated dermatomyositis, can present with normal CK levels [16,17].

Standard management involves discontinuing statins and initiating systemic corticosteroids as first-line therapy [18]. Consequently, alternative approaches for long-term lipid management are required. Proprotein convertase subtilisin/kexin type 9 (PCSK9) inhibitors have emerged as a promising, guideline-recommended alternative for these patients. Current evidence suggests that PCSK9 inhibitors are safe for individuals with statin-associated muscle symptoms (SAMS), including severe forms such as rhabdomyolysis, and do not appear to carry the same risks of new-onset diabetes or cognitive side effects. While further long-term studies are warranted, they represent a key therapeutic choice for high-risk patients who cannot tolerate statins.

For our patient, who was using statins prophylactically, we advised lifelong abstinence from this class of medication. Because symptoms of SINAM typically recur upon rechallenge, his long-term hyperlipidemia care will focus on dietary modification, weight reduction, and other non-statin agents [18]. Prognostically, factors such as male gender and the concurrent use of multiple immunotherapies are associated with favourable outcomes in NAM, which is a relevant consideration for this patient's long-term management [4].

Patient perspective

The patient's wife shared their immense relief and gratitude regarding his recovery. 'Five weeks ago, he was completely bedridden,' she recalled. 'I never could have anticipated such a remarkable turnaround. Now, he's back to his usual self, walking and climbing stairs, and we are even planning a holiday.' They expressed how frightening the experience was and how grateful they were for the diagnostic shift that led to the correct treatment.

## Conclusions

This case highlights the significant diagnostic challenge of differentiating a systemic autoimmune process from infection in a complex postoperative patient. It highlights the importance of considering SINAM in patients presenting with progressive proximal muscle weakness and elevated CK levels, particularly when symptoms persist or develop despite discontinuation of statin therapy. As SINAM can have a delayed onset, occurring months or even years after initial statin exposure, a thorough and detailed medication history remains crucial. Importantly, this case also highlights the value of an orthogeriatric approach. In a younger, otherwise healthy patient, extensive investigations, such as muscle biopsy and comprehensive autoantibody panels, might have been pursued without hesitation. However, in older adults, particularly postoperative patients with reduced physiological reserve, prolonged hospitalization, and increased risk of hospital-acquired complications, a more holistic, clinically guided strategy is essential. Prioritizing early treatment based on clinical suspicion, rather than delaying care for confirmatory investigations, can prevent deterioration and support recovery.

Furthermore, this report reinforces two key clinical principles. First, a negative serological result does not exclude the diagnosis; in such cases, clinical judgment, supported by the patient’s presentation and treatment response, must guide decision-making. Second, the rapid and marked clinical improvement observed following corticosteroid initiation emphasises the importance of timely intervention to prevent long-term disability. Given the risk of recurrence with re-exposure, lifelong statin avoidance is imperative. In conclusion, this case illustrates the need for a high index of suspicion for SINAM in the appropriate clinical context. Recognizing the characteristic features and acting decisively, especially in vulnerable older adults, can dramatically alter the disease course, enabling recovery and preserving function.
